# Supplementary material for: Microevolution, reinfection and highly complex genomic diversity in patients with sequential isolates of Mycobacterium abscessus
Source: Nat Commun. 2024 Mar 28;15:2717. doi: 10.1038/s41467-024-46552-w (PMC10979023; doi:10.1038/s41467-024-46552-w)
Supplement: Supplementary file 1 — Supplementary Information [file 41467_2024_46552_MOESM1_ESM.pdf]

Supplementary Table 1. Pairwise genomic distances (SNVs) among all patients with:

- *Mycobacterium abscessus subsp. abscessus*

	Patient1	Patient2	Patient4	Patient5	Patient7	Patient8	Patient10	Patient11	Patient12	Patient13-1	Patient13-2	Patient14
**Patient1**	-	19811	22199	20330	28260	19802	19417	22195	19816	21252	19811	19862
**Patient2**	19811	-	17584	15603	23767	30	16316	19924	145	15959	82	201
**Patient4**	22199	17584	-	17979	26009	17575	18834	20952	17513	18381	17584	17561
**Patient5**	20330	15603	17979	-	22868	15591	16329	17841	15531	15264	15603	15584
**Patient7**	28260	23767	26009	22868	-	23760	24765	26133	23700	22514	23771	23748
**Patient8**	19802	30	17575	15591	23760	-	16307	19915	132	15946	69	188
**Patient10**	19417	16316	18834	16329	24765	16307	-	16582	16333	16855	16317	16383
**Patient11**	22195	19924	20952	17841	26133	19915	16582	-	19913	18749	19928	19961
**Patient12**	19816	145	17513	15531	23700	132	16333	19913	-	15886	143	112
**Patient13-1**	21252	15959	18381	15264	22514	15946	16855	18749	15886	-	15956	15932
**Patient13-2**	19811	82	17584	15603	23771	69	16317	19928	143	15956	-	199
**Patient14**	19862	201	17561	15584	23748	188	16383	19961	112	15932	199	-

The first isolate of each patient is included, except for the case with a reinfection (Patient 13)

- *Mycobacterium abscessus subsp. massiliense*

	Patient3	Patient6	Patient9
**Patient3**	0	30521	30515
**Patient6**	30521	0	34
**Patient9**	30515	34	0

The first isolate of each patient is included

Supplementary notes

Therapy information

The patient was a 35-year-old woman with severe homozygous F508del cystic fibrosis, diagnosed at 2 years of age with pulmonary, digestive, and hepatic manifestations, and exocrine and endocrine pancreatic insufficiency. In brief, in May 2007, the patient was treated with azithromycin. In September 2007, with the initiation of *M. abscessus* treatment, azithromycin was changed to intravenous amikacin (5mg/kg/day) and cefoxitin (2g) for a month, together with clarithromycin (500mg BID), which was maintained. In February 2009, with the isolation of a new *M. abscessus*, treatment was changed to clarithromycin, levofloxacin (500mg every day), and trimethoprim-sulfamethoxazole (160mg/800mg BID). In May 2010, levofloxacin and trimethoprim-sulfamethoxazole were discontinued, and clarithromycin was continued, reinforced for one month by intravenous amikacin and cefoxitin. After the reinforcement, the patient was treated with levofloxacin and trimethoprim-sulfamethoxazole. In February 2011, active treatment was discontinued, and clarithromycin was maintained until December 17, 2019, when it was discontinued and replaced with azithromycin every 48 hours (Figure 2). In 2022, it was decided to maintain treatment with no further attempts at eradication. The treatment protocol consisted of inhaled aztreonam, alternating every 14 days with inhaled levofloxacin and azithromycin.

From July 2008 to September 2019, the patient received inhaled colistin, which was then discontinued, and inhaled tobramycin was initiated. However, the patient showed very poor tolerance to inhaled tobramycin and received it erratically from September 2019 to March 2021. From September 2015 to November 2020, inhaled aztreonam was also administered, but was stopped due to poor tolerance. In March 2021, the patient resumed inhaled aztreonam treatment in 28-day on/off cycles. Along with inhaled aztreonam, oral levofloxacin was initiated in March 2021, with a treatment cycle of 28 days. Oral administration continued until September 2021 when it was switched to inhaled levofloxacin, which is currently ongoing. The patient also started treatment with CFTR modulators with tezacaftor/ivacaftor (Symkevy) in March 2020. In January 2022, the patient discontinued tezacaftor/ivacaftor and started treatment with elezacaftor/tezacaftor (Kaftrio). The patient has been receiving the latter treatment since then, and continues to do so (Figure 2).

Supplementary Table 2. Pairwise genomic distance of the different genotypes of patient 14, based on high-quality unique SNV calls (>20X depth and >0.8 allelic frequency)

	Genotype 1	Genotype 2	Genotype 3	Genotype 4	Genotype 5	Genotype 6	Genotype 7	Genotype 8	Genotype 9	Genotype 10	Genotype 11	Genotype 12
**Genotype 1**	-	76	172	164	219	194	258	196	198	254	319	171
**Genotype 2**	76	-	156	88	143	118	242	120	182	178	243	155
**Genotype 3**	172	156	-	244	299	274	274	276	214	334	399	187
**Genotype 4**	164	88	244	-	231	206	330	208	270	266	331	243
**Genotype 5**	219	143	299	231	-	247	385	263	325	307	372	298
**Genotype 6**	194	118	274	206	247	-	360	238	300	60	145	273
**Genotype 7**	258	242	274	330	385	360	-	362	300	420	485	273
**Genotype 8**	196	120	276	208	263	238	362	-	302	298	363	275
**Genotype 9**	198	182	214	270	325	300	300	302	-	360	425	55
**Genotype 10**	254	178	334	266	307	60	420	298	360	-	205	333
**Genotype 11**	319	243	399	331	372	145	485	363	425	205	-	398
**Genotype 12**	171	155	187	243	298	273	273	275	55	333	398	-

Supplementary Table 3. List of genes involved in hypermutator phenotypes in mycobacteria which have been studied to search for SNVs.

GENOME	START	END	GENE
**CU458896.1**	207716	208180	AhpD (peroxidase) [MAB_0197]
**CU458896.1**	417805	418602	endonuclease III [MAB_0418]
**CU458896.1**	559115	559957	mutY [MAB_0558c]
**CU458896.1**	696619	697107	peroxidase [MAB_0693]
**CU458896.1**	702205	703212	peroxidase [MAB_0700c]
**CU458896.1**	1061713	1064136	pcrA/uvrD1 [MAB_1054]
**CU458896.1**	1176042	1177289	peroxidase [MAB_1160]
**CU458896.1**	1177295	1178554	peroxidase [MAB_1161c]
**CU458896.1**	1295175	1295669	Tpx (peroxidase) [MAB_1290c]
**CU458896.1**	1308955	1309347	mutT2/NUDIX (hydrolase) [MAB_1308]
**CU458896.1**	1470132	1470830	nucS [MAB_1460]
**CU458896.1**	1535314	1535799	mutT/NUDIX [MAB_1514]
**CU458896.1**	2096195	2096662	mutT/NUDIX [MAB_2096c]
**CU458896.1**	2317057	2318265	peroxidase [MAB_2270c]
**CU458896.1**	2421817	2422461	mutT/NUDIX [MAB_2365]
**CU458896.1**	2522494	2524710	katG (catalase/peroxidase) [MAB_2470c]
**CU458896.1**	2757356	2759635	mutB [MAB_2711c]
**CU458896.1**	2759637	2761460	mutA [MAB_2712c]
**CU458896.1**	3063170	3063622	dut [MAB_3003c]
**CU458896.1**	3278595	3279563	fni [MAB_3242]
**CU458896.1**	3280257	3281021	[MAB_3244]
**CU458896.1**	3294595	3295455	mutM [MAB_3255c]
**CU458896.1**	3321845	3322525	ung [MAB_3283c]
**CU458896.1**	3330283	3331218	mutT/NUDIX [MAB_3291]
**CU458896.1**	3380937	3382436	gatB [MAB_3334c]
**CU458896.1**	3509847	3510272	mutT/NUDIX [MAB_3469]
**CU458896.1**	3520353	3521018	[MAB_3480]
**CU458896.1**	3521105	3522853	fadE [MAB_3481]
**CU458896.1**	3552723	3554792	uvrD [MAB_3511c]
**CU458896.1**	3557288	3560524	uvrD-like helicases [MAB_3515c]
**CU458896.1**	3560521	3563697	uvrD-like helicases [MAB_3516c]
**CU458896.1**	3630794	3631216	[MAB_3581c]
**CU458896.1**	3755926	3759162	dnaE2 (error-prone DNA polymerase) [MAB_3703c]
**CU458896.1**	3960754	3962556	peroxidase [MAB_3909]
**CU458896.1**	3992388	3992846	peroxidase [MAB_3943]
**CU458896.1**	4104283	4105614	mshA [MAB_4057c]
**CU458896.1**	4205843	4207423	[MAB_4141]
**CU458896.1**	4278712	4279428	mutT/NUDIX [MAB_4210]
**CU458896.1**	4288120	4288668	mutT3 [MAB_4221c]
**CU458896.1**	4422631	4423068	mutT/NUDIX [MAB_4342c]
**CU458896.1**	4486907	4487434	AhpD (peroxidase) [MAB_4407c]
**CU458896.1**	4487450	4488037	peroxidase [MAB_4408c]
**CU458896.1**	5003633	5005009	Mtu DnaB (endonuclease III) [MAB_4895c]
**CU458896.1**	5044671	5045486	mutT/NUDIX [MAB_4935]
**CU458896.1**	3109126	3109740	RecX (dna repair) [MAB_3059c]
**CU458896.1**	3109706	3110746	RecA (dna repair) [MAB_3060c]
**CU458896.1**	1513875	1514561	dna repair [MAB_1500]
**CU458896.1**	1898460	1899110	dna repair [MAB_1901c]
**CU458896.1**	1042671	1044923	dna repair [MAB_1033]
**CU458896.1**	2655155	2657863	DNA polymerase (POL1) [MAB_2615c]
**CU458896.1**	2385436	2386917	AlkA (dna repair) [MAB_2333c]
**CU458896.1**	2407352	2407969	dna repair [MAB_2352]
**CU458896.1**	1349273	1349851	TagA (dna repair) [MAB_1349]
**CU458896.1**	1605050	1605847	Endonuclease VIII (dna repair) [MAB_1575]
**CU458896.1**	4840829	4841593	Endonuclease VIII (dna repair) [MAB_4728c]
**CU458896.1**	1047655	1048518	dna repair [MAB_1038]
**CU458896.1**	278241	279272	LigC (dna repair) [MAB_0279c]
**CU458896.1**	109422	110027	Ku domain protein (dna repair) [MAB_0108c]
**CU458896.1**	1041773	1042582	Ku70/Ku80 (dna repair) [MAB_1032c]
**CU458896.1**	2689974	2690417	mmpS1 (eflux pump, enhance growth within macrophages) [MAB_2649]
**CU458896.1**	3587911	3588618	RNA polymerase sigma-E factor [MAB_3543c]
**CU458896.1**	3587606	3587914	Conserved hypothetical protein (possible anti-sigma factor) [MAB_3542c]
**CU458896.1**	3473766	3474317	Probable RNA polymerase sigma-C factor [MAB_3428c]
**CU458896.1**	3080141	3081112	RNA polymerase sigma factor [MAB_3028]
**CU458896.1**	1359744	1360514	Probable alternative RNA polymerase sigma factor [MAB_1362]
