# Supplementary material for: Microevolution, reinfection and highly complex genomic diversity in patients with sequential isolates of Mycobacterium abscessus
Source: Nat Commun. 2024 Mar 28;15:2717. doi: 10.1038/s41467-024-46552-w (PMC10979023; doi:10.1038/s41467-024-46552-w)
Supplement: Supplementary file 3 — Description of Additional Supplementary Files [file 41467_2024_46552_MOESM3_ESM.pdf]

<u>Description of Additional Supplementary files</u>

Supplementary Data 1: Expanded analysis of the SNPs identified between sequential isolates from each patient in which we detected intrapatient microevolution leading to acquisition of diversity.
